# Preparedness, resilience and unmet needs of informal caregivers of advanced cancer patients in a Regional Mission Hospital in Kenya: Qualitative Study

**DOI:** 10.1186/s12904-022-01048-6

**Published:** 2023-02-28

**Authors:** Wesley Too, Faith Lelei, Mary Adam, Pete Halestrap

**Affiliations:** 1 Aga Khan University, University Center, P O Box 30270-00100, 5th Floor, SONAM, South Wing, 3rd Parklands Avenue, Off Limuru Road, Nairobi, Kenya; 2 AIC Kijabe Mission Hospital, Nairobi/Naivasha Rd, Karuri, P O Box 20-00220, Nairobi, Kijabe Kenya

**Keywords:** Informal caregiver, Advanced cancer, Family caregiver, Qualitative, Experiences, End-of-life

## Abstract

**Background:**

Cancer is the third highest cause of death in Kenya. Eighty percent of cancer cases arrive at advanced stages, when there is nothing that can be done to cure them, and palliative care is the best alternative. Although the majority of end-of-life care in Kenya is provided at home, little is known about the caregivers’ preparedness, resilience and continued unmet needs. The goal of this qualitative study was to explore caregivers’ perceived preparedness, resilience and continued unmet needs in their caregiving role to patients with advanced stages of cancer.

**Methods:**

A purposive sampling method was used to identify and recruit twelve informal, home-based caregivers of patients with advanced cancer from Kijabe Palliative Clinic data base. Interviews were conducted in patients' homes. The data was analyzed using interpretive phenomenological analysis approach. Ethical considerations were observed. Participants were kept anonymous and confidentiality.

**Results:**

Competing tasks, lack of preparedness in handling end-of-life care for patients in advanced stages of cancer were the main concerns. Continued unmet needs and financial stresses, and vulnerability for female caregivers all contributed to increased caregiver burden in this study. Caregivers were however determined and resilient amidst challenges that faced them, they exhibited hope against hopelessness. Some caregivers were vulnerable and faced potential for abuse following anticipated loss of their family member exacerbated psychosocial stress and needs

**Conclusion:**

Informal caregivers had common unmet needs related to caring for their advanced cancer patients. Whilst family caregivers had huge caregiver burden, insurmountable practical challenges related to role overload and competing tasks, they remained resilient though unprepared in giving end of life care.

**Recommendations:**

Caregivers should also be examined, prepared, and supported during clinic reviews. More research is needed on the use of telephones for caregiver follow-up, the impact of introducing caregiver-targeted screening tools on caregiver quality of life and their impact on enhancing caregiver well-being in order to prepare & support them adequately for the caregiving role.

## Introduction

Informal caregivers have been known to shoulder responsibilities of caring for patients with advanced cancer in sub-Saharan Africa due to paucity of institutional facilities. Previous studies show that factors associated with caregiver burden include previous hospitalisation of the care recipient and perceived dysfunction in patients’ activities of daily living [1]. Cancer care has shifted from in-patient to ambulatory and home settings as a result of changes in the health-care system. This transition has also resulted in more family involvement in the day-to-day care of the cancer patient. Patients with cancer require a variety of services, including disease and treatment monitoring, symptom management, medication administration, emotional support, personal care assistance, and instrument care assistance [2]. Informal caregivers may be unprepared to take on these responsibilities, which necessitates knowledge of the condition and its treatment, as well as training in technical and caring skills. In addition, caregiving must be balanced against pre-existing roles and duties [3]. Furthermore, informal caregivers may require coaching and emotional support as a result of their own emotional reactions to the patients' diagnosis and prognosis [4].

It is widely recognized that informal caregivers are not adequately prepared to handle end-of life-care for patients with advanced cancer [5, 6]. While caregivers are reported to be resilient, they face complex intricacies which can negatively impact their health in various domains; physical, psychological, emotional and spiritual. The impact manifests in various domains; physical, psychosocial and emotional aspects of their lives.

Resilience among many informal caregivers of patients with advanced cancer has been suggested as a primary component in their ability to withstand psychological strain and function effectively in the task despite carrying a heavy load of caregiving responsibilities [3, 7]. Whilst studies examining resiliency and preparedness of informal caregivers of advanced cancer patients reported higher resilience associated with higher caregiver preparedness, higher readiness for surrogate decision-making and lower anxiety and depressive symptoms [3, 8, 9], there is less insight into Kenyan experiences of informal caregivers of advanced cancer patients. This is particularly important since studies have shown that resilience may be critical to informal caregivers' abilities to manage stress, be effective sources of support to patients, and feel ready to make future medical decisions on behalf of patients [3, 7].

Studies have shown that caring for advanced cancer patients might have a negative impact on one's health [1, 10, 11]. Because of the intensive nature of illness and treatment, cancer informal caregivers confront particular obstacles, which raises their risk of burden, low quality of life (QOL), and burnout. Whilst these trend increases informal caregivers’ unmet needs across all spheres of life, there is less-developed knowledge on the insights of caregivers of advanced patients with cancer on a follow-up palliative care in a mission hospital.

Informal caregivers are reported to provide significant care for patients with advanced cancer while also suffering from concealed morbidity and unmet demands and needs. A study examining risk factors associated with caregiving for patients with advanced cancer report pertinent interventions and provide a practical framework for palliative care of informal caregivers. Interventions recommended include assessing the caregiver's situation and needs such as referring to appropriate services and resources which is critical for wellbeing of caregiver [12]. Educating about practical aspects of caregiving and supporting informal caregivers through bereavement is equally important.

A study assessing unmet needs of patients living with advanced cancer and their informal caregivers report significant challenges, including persistent physical symptoms and psychosocial concerns, difficulties with coordination of care, and possible lack of available resources and supports if the person is no longer being followed by cancer health care professionals [2]. In a study to estimate the prevalence rate of depression in cancer patient informal caregivers and to identify factors affecting depression and quality of life of cancer caregivers found out that a considerably high prevalence of depression is found in cancer patients’ caregivers. Several factors affecting depression and their quality of life of cancer patient caregivers have been documented [13]

A study assessing the effect of cancer patients' and their informal caregivers' physical and emotional symptoms on caregiver burden, revealed increased caregiver burden in family caregiver influenced by interplay of patients' and their own symptoms and problems [10]. For instance, there were significant associations between caregiver burden and the patient-related variables such as self-efficacy, sleep disturbance, social support, fatigue and symptoms. Further, gender differences have been linked to differences in caregiver stress, burden, anxiety, depression, and coping styles, as well as how caregiver’s gender impacts patient outcomes in the context of advanced cancer patients [10, 14]. It is reported that female informal caregivers had significantly higher levels of perceived stress, depression, anxiety, and social strain compared with male caregivers [15]. These findings highlight the potential differences between male and female caregivers' needs and psychological health.

The caregiver burden and continued unmet needs have been shown to have ill effects on the health of the informal caregivers: physically, emotionally, mentally and psychologically. Whilst evidence-based caregiver interventions are well documented [16], the informal caregivers’ unmet needs, the effects they experience as a result of caregiving, and potential resources and sources of support largely remain unknown in the East African setting [17]. There is however growing research interest in end-of-life care for patients with advanced cancer and informal caregivers’ experiences in this region. Whilst informal caregivers for advanced cancer patients are a fundamental source of care for cancer patients in Kenya, the population of informal caregivers and their tasks, psychosocial needs, preparedness, and resilience are not well understood. This study therefore seeks to explore experiences of informal caregivers focusing on preparedness, unmet needs and resilience among informal caregivers of patients with advanced cancer patients following referral to one of the palliative care units in a mission hospital in Kenya

### Study design

Using a phenomenological qualitative approach, participants of the study were recruited from palliative care database of end-stage patients on home visitation from Kijabe Hospital (palliative care clinic) in Kenya.

### Study setting

Kijabe Hospital runs an outpatient palliative unit among other services. It took care of approximately 1100 palliative care patients. Eighty percent of these patients had cancer, and almost all of them received their end-of-life care at home.

### Population

As recommended by the interpretative phenomenological analysis (IPA) [18], only a small number of participants meeting inclusion criteria.

### Recruitment strategy

Self-identified informal caregivers of adult patients with advanced cancers (stage 3 or 4) who are legal adults (over age 18) and understand English or Kiswahili were recruited. The informal caregivers cared for patients who have a performance status of 3 or 4 according to the WHO performance staging (i.e. patients who spend more than 50 % of their waking hours in bed, or completely bedridden, and capable of limited or no self-care.). Twelve informal caregivers of advanced cancer patients were purposefully selected for this study and the in-depth interviews were done in the patients’ home for the patient’s and the caregiver’s convenience. Once identified and agreed upon, the palliative team contacted the patient or caregiver to establish their availability for a home visit. This initial contact was made via a telephone call. If the patient and caregiver were available for the home visit, the researcher then sought preliminary consent via telephone to do the study in addition to the intended home visit.

### Sample

To further understand the experiences, twelve semi-structured interviews from informal caregivers’ preparedness, unmet needs and resilience in cancer caregiving were explored. We chose nine interviews from the interview data that were the richest in terms of our research goal and in accordance with the guidelines for interpretative phenomenological analysis methodology [18]. The ninth interview, though, appeared to decipher a new code. Three further interviews were chosen as a result. Despite this, no additional codes could be found, leading one to believe that data saturation had been reached with this sample.

### Data collection

The purpose of the interview guide was originally to investigate the preparedness of informal caregivers to give care at end-of-life, explore unmet needs and examine resiliency in cancer caregiving. Questions about informal caregivers' experiences providing informal care were added to the initial interview guide for the study's purposes. The dataset for the current study was created from these interview segments as well as those where individuals spontaneously discussed their experiences giving care.

### Ethical considerations

The researcher informed the caregiver and family about free, voluntary participation in the study and that should they not wish to participate, the home visit by the palliative team would carry on as planned. Once preliminary consent was obtained, the home visit was scheduled. The researcher and one nurse from the palliative team would go for the visit. During the home visit, the researcher obtained written consent. No caregiver or patient refused to take part in the study. Ethical approval was obtained from the Research and Ethics Committees of both Kijabe Hospital, Kabarak University Research Ethics Committee (KABU/IREC/18/003) and National Commission for Science, Technology, and Innovation (NACOSTI) -NACOSTI/P/19/75630/27563. The researcher obtained written consent from the caregiver at the patients’ home before proceeding to conduct the interviews. Confidentiality and anonymity were ensured by using codes

### Data analysis

According to the process outlined by an interpretative phenomenological analysis [19], twelve interviews were inductively evaluated. The interviews were read multiple times, and three levels of interpretation—descriptive (remaining true to the text), linguistic (exploring language use), and conceptual (understanding the participant's concerns]—were remarked on. The authors' team discussed the narrative summaries of each participant's stories. An Interpretative Phenomenological Approach was used to examine the interviews in a hermeneutic manner. In a hermeneutic analysis, the researcher cycles back and forth over the data, evaluating each interview question within the context of the participant's narrative and taking into account how the context is in turn influenced by the many interview questions [18]. After the interviews were coded, trends were found both within the interviews (in-case analysis) and across interviews using an iterative technique (across-case analysis). As a result, the codes were developed and associated with quotes. The codes were then organized into clusters of topics, and the themes into concepts. The research team deliberated each topic and concept's applicability to the study issue until agreement was obtained [18]. The ideas and meanings based on the complete dataset were eventually produced, discussed, and polished using a hierarchical map.

#### Trustworthiness and credibility

Four approaches as described by Guba & Lincoln [20] were followed to ensure that the qualitative data was credible. Researchers enhanced confirmability, transferability, dependability and utilized reflexivity to improve the interviewer's trustworthiness and dependability. To ensure reflexivity, field notes were taken both during and right after each interview. The analysis was carried out in a controlled and traceable manner to assure trustworthiness and credibility, and a collaborative interdisciplinary team used in-case and across-case assessments to confirm the themes' applicability.

#### Findings

A total of twelve [12] informal caregivers were interviewed. Their sociodemographic characteristics are shown on Table 1

**Table 1:** Socio-demographic characteristics of informal caregivers

	Patient age and Gender	Cancer	Caregiver age and Gender	Relationship	Duration of caregiving
1	79, M	Prostate cancer with spine Mets	53, M	Son	5 months
2.	75, M	Prostate cancer with spine Mets	21, M	Grandson	1 year
3.	89, M	Metastatic pancreatic cancer	41, F	Daughter	4 months
4.	32, F	Metastatic Gastric cancer	26, F	Sister	1 year
5.	63, F	Esophageal Cancer	67, M	Husband	16 months
6.	40, M	Cholangiocarcinoma	37, F	Wife	10 months
7.	84, F	Metastatic Lung Cancer	42, F	3^rd^ Wife	8 months
8.	75, M	Prostate cancer with spine Mets	18, M	Grandson	18 months
9.	60, F	Cervical Cancer	62, F	Sister	14 months
10.	67, F	Esophageal Cancer	35, F	Daughter in law	2 years
11.	77, F	Cholangiocarcinoma	41, F	Daughter	1 year
12.	66, M	Duodenal Cancer	61,F	Wife	10 months

#### Preparedness of informal caregivers

This study reports several informal caregivers who found themselves very vulnerable and ill-prepared or trained to take care of their loved ones with advanced Cancer. In one interview, a 62-year-old female caregiver caring for her sister with advanced cancer patient reports overwhelming care burden concerns which have impacted on psychological well-being. Another 26-year-old reports of caring for their family member (sister) with advanced Gastric cancer with no prior training or preparedness, but simply offering care:

“One thing that would really help me is if you had someone to talk to me, and counsel someone on how I can stay with someone who is sick, so that even if someone is overwhelmed by the disease, I can know how to handle her. That counselling would be very important.” CG9, 62-year-old sister

“I have not gotten any training to take care of her. It just comes from my willingness” CG4, 26-year-old Sister

Among other informal caregivers, they found caregiving experiences most difficulty for those who had no prior preparations. For example, as it draws towards end of life of their family member, a 42-year-old female caring for an advanced lung cancer patient reports having to simply wait for any eventuality. Majority informal caregivers reported state of helplessness especially when one may not know, one may not know exactly what happens at what stage and what to expect. In one other case, a 41-year-old female caregiver caring for advanced pancreatic cancer patient reported lack of knowledge on what to do next for their patient and stated that so and state that she simply does what she knows best. Another 26-year-old female caring for a sister with advanced gastric patient expresses psychosocial effects of caring for a patient at end of life:

“The stress now is you know we have been explained to that there is no treatment. She cannot be treated again. Now the stress is there because you know its any time….because now if the patient is not being treated and is physically ill, what would you conclude for yourself?..... and even sometimes when she lacks sleep she tells God to come and take her now…..” CG4, 26-year-old Sister

“Now if somebody is like this, and you know the kind of ailment he has, you just wait for anything. You have to be ready for anything.” CG7, 42-year-old wife

“You see my sisters, they are far away. You know I cannot always rely on them….. and at other times I do not know what to do….. I haven’t been taught how to care for him, but I just look and try and see whats the best thing. CG3, 41-year-old daughter

In some cases, basic activities of daily living such as feeding and toileting were difficult for the patients, and this posed significant challenges for the caregiver. One caregiver 35 year old female taking care of advanced esophageal cancer report expresses her insight on her experiences of their patient with stoma:

“I understand why she does not eat- I was told there’s no way for the food to pass through. But my elder siblings don’t understand. They do not understand because they have not been bringing her to hospital…”CG10, 35-year-old daughter-in-law

“He cannot sit in a basin, and we don’t have money for diapers. So we put him on top of a bucket that is covered. And we have made a cut on the middle of its top….thats where he sits on to pass stool.” CG12, 61-year-old wife

From this study, no family had sufficient financial reserves to adequately meet the unexpected costs. As such, most families had depleted their savings and resources and were getting poorer with financial demands each day. From caregiver’s experiences, it portrayed a picture and state of hopelessness resulting from resource-draining venture as money is quite spent shuttling between hospitalizations and home as well as buying medications. One caregiver, 18-year-old grandson taking care of grandfather with metastatic lung cancer was frightened that their residence could be auctioned for lack of money to repay loans taken to treat their family member. Another 37-year-old caregiver of an advanced Cholangiocarcinoma patient reported seeking help from neighbourhood to meet financial demands related to medical care:

“We have difficulty in getting the pain killers, and the difficulty is financial. Sometimes we have to sell some sheep to get money for the medicine…. My grandmother and I are even so scared that this place may one day be auctioned…..Grandma has borrowed a lot of loans ” CG8, 18-year-old grandson

“I loved him, but I did not have the money. I did not know what to do . I talked to the villagers and his uncles. CG6, 37-year-old wife

### Resilience

Almost all the informal caregivers were hopeful about recovery for their patients. To put this in context, the participants of the study were patients who had advanced cancer, with a WHO staging of 3 of 4 (patients who spent more than 50% of their waking hours in bed or completely bedridden, and capable of limited or no self-care.) Within a month of the interviews 8 out of 12 had died, including those excerpts are below, died within one month of conducting interviews, and in 3 months, all the patients had died. This hope for recovery is also shared by the church sometimes and perpetuated by it. One 21-year-old respondent said:

“So you see I’d rather stay here helping him until he gets well” CG 2, 21 year old grandson

Another caregiver taking of her sister reported:

“There is a day she (the patient) said she will die, I told her no, she will not die but live… I still pray for L to get well, so that she can bring up her children because I cannot bring up her children the way she would have… It's better for her to stay here with us as long as we are able to see her, even if she remains weak… I want her to recover... the church members come to pray for her, and tell her that she will recover” CG 4, 26-year-old sister

As such, given the lack of acceptance of death it was a challenge for the interviewer to explore end of life symptoms and management.

Resilience and resolute caregiving despite a challenging environment and circumstances was reported among almost all of the informal caregivers of advanced cancer patients. Some informal caregivers though were on the verge of giving up caregiving due to deplorable and psychological stressful states of conditions of caregiving, but others drew strength intrinsically through prayers. To some, they drew strengths intrinsically through prayers to God. A 41-year-old daughter prays for God’s strength to keep caring for their mother who has advanced pancreatic cancer and as does a 35-year-old daughter in law who prays for God’s intervention:

“Psychologically, I have been affected because I wonder what next. I wonder when I have gone to work and left him, will I find him? When I find him in the bedroom, I tell God thank you ….. I cannot accept to be affected physically, because I will not be able to care for him when I am affected CG3, 41-year-old daughter

“I don’t want to think too much. I can start putting a heavy burden on myself that I cannot carry. I just pray to God for help with my thoughts to go away.“ CG10, 35- year-old daughter in law

Similarly, others were determined in their caregiving in honor of their marital vows. Whilst others religiously devoted to care out of love for their family member (s) with advanced cancer patients. Others reported of their undying commitment to support their patient through most difficult and challenging times. One spousal-caregiver reports of her experiences:

“You know there is one thing: on our wedding day, we said this- in the day of trouble I will look after him, I’ll be with him. Now this time if I leave him on the day he’s in trouble, really, do I fulfill the word of God? So I will just stay and serve him until the last day.” CG12, 61-year-old wife

“There is a day she said she was about to die, I told her that she will not die but that will live and take care of her children ….I just pray that God my heal her so that she may raise her children CG4, 26 year old sister

Resilient informal caregivers endured these strains at great personal sacrifice and cost. Some felt it was their duty because this was their parent or grandparent. Two of the spouses felt that it was their obligation because of the vows they made to each other when they got married several years ago to care for each other in sickness and in health. Those nursing their siblings said they did it out of the love that they had for them. Two informal caregivers left their matrimonial homes to care for their loved ones; one with the blessing of her husband and the other with resistance from her husband. Both left their homes with the expectation to return to their homes once their services were no longer needed. Some informal caregivers even did duties that were culturally and traditionally not perceived to be theirs, sometimes even with cultural breaches.

One respondent aged 41 who left her matrimonial home to care for her mother said:

“I told him that he can always get another wife, but I cannot get another mother. So I am going to take care of my mother whether he likes it or not …” CG 3, 41 year old daughter

Another caregiver (husband) taking care of his wife who had advanced esophageal cancer reported:

“I have no maid, I don’t have any money to employ one…. I cook for her; I boil for her drinking water …. I do her laundry” CG5, 67-year-old husband

Another caregiver (grandson) aged 18 taking care of his grandmother who was paraplegic and his grandfather who was also paralyzed secondary to metastatic prostate cancer reported:

“She just has different issues, for example, if she needs to relieve herself, I take her to the toilet, she just goes there and sits. When she is done, I go for her …” CG 8, 18-year-old grandson

Others were determined to carry out their caregiving responsibilities by whichever means possible. A 53-year-old son caring for his father with advanced prostate cancer provided support against backdrop of lack. Another caregiver, 41-year-old daughter left her marital home to take care of her father with advanced pancreatic cancer. 61-year-old wife taking care of her husband with advanced duodenal cancer continued to give care and support relentlessly:

“If I could have 2 or 3 hours a day to do my own work, it would really help me financially. For now I cannot depend on anyone in the family I have to make sure he has eaten and taken his medication. So I cant go out for long …..” CG1, 53-year-old son

I had been married, but I came back home to care for him CG3, 41-year-old daughter

“I have tried to leave the stress alone. Because even if I stress myself, and he is already unwell, what I am going to do? I will be sick and now who will look after us?” CG12, 61-year-old wife

Resiliency was also evident among some informal caregivers who when faced with state of hopelessness, they did not give up instead, they found coping strategies and pushed on with their caregiving. Though some reported difficulties in care-giving, they overcame all odds and supported their family member(s) suffering from advanced cancer.

“I don’t think I have been affected taking care of mum. And even though it had, I wouldn’t know because I cant see or feel.” CG11 –41 year old daughter

“There is a day she said she was about to die, I told her that she will not die but that will live and take care of her children ….I just pray that God my heal her so that she may raise her children” CG4 26 year old sister

“ I was mocked a lot by people, saying I would not succeed in fundraising money to take him to hospital. I was tolerating all these because I had hope but he had given up hope. He even asked me to look for village elders so that he may talk to them about final rites but I refused…..he said to me “ I love you all but my life has to me to an end” but I told him there is a God in heaven who will help you” CG6 37-year old wife

“But when I tell them that they think am young and ignorant, and they disregard me … … because you just can’t watch as a family disintegrates. Its just very sad”CG8 18 years old grandson

Other informal caregivers have sacrificed a lot in their support. For instance, a 21-year-old son taking care of advanced prostate patient has sacrificed a lot to get expensive medication not found within the region they live. Same with 18-year-old grandson taking care of a patient with metastatic lung cancer:

“Sometimes it’s a bit difficult to get medicine, like this called Bicalutamide is hard to get it. There is no place to get it here in the rural areas, so we have to go to Nairobi to get it. And its expensive….” CG2 21-year-old grandson

“My farm is just bare, yes even here there’s one of my own that is not ploughed, because of the work of taking care of him….. I even had a hotel I was operating, hotel business is good, but I had to stop it” CG8 18-year-old grandson

Despite lack of support from some family members and environment and lack of financial resources, some caregivers struggled amidst quite enormous challenges to provide for their patients’ basic needs such as food. Another caregiver hoped to recover what was lost during caregiving process:

“When she was brought to my place, everyone left and went to their own homes. The burden was left with me. That’s what makes caring for her hard sometimes ….. I notice the food that she eats, and sometimes she needs something better, and I have to buy it. The informal caregivers will come to greet our grandmother empty handed, they do not know what she has eaten …..” CG9 62-year-old sister

“When they are better, I want to start a new life. I just want to re-establish my business, same to my farms.” CG8 18-year-old grandson

### Unmet Needs

Informal caregivers continue to face unlimited challenges in their caregiving including unmet needs at individual-level. Some of their unmet needs include lack of network of support, psychological impact of caregiving with no resources to support them, helplessness related to hopeless situation or state of caregiving. Some informal caregivers face myriads of challenges related to their growing unmet needs:

“The one thing that would have helped me to care for my grandparents, is a house helper. So that she can look after one person, and I look after another. Because looking after these two is hard ………….. Just remembering my stuck projects, I get very stressed

CG8 18-Year-old grandson

“My children are not financially capable, so they have to rely on me. And I still have to take care of my wife. So my main challenge now is financial. And I don’t have a house help to care for her, I do not have the money to hire one CG5 67-year-old-wife

“My farm is just bare, yes even here there’s one of my own that is not ploughed, because of the work of taking care of him….. I even had a hotel I was operating, hotel business is good, but I had to stop it”CG8 18-year-old grandson

Burn-out resulting from increased care-burden with no where else to turn to contributed to role-strain and inability to cope with caregiving. There was evident physical impact of relentless caregiving which manifested in lose of weight of some informal caregivers to stressful conditions of caregiving affecting their eating & rest habits:

“There was a time he could not turn himself on the bed. You are the one to turn him. Sometimes I could talk to him but he isn’t talking. It was hard…… I thought he was now going.” CG7 42-year-old third wife

When we both went to hospital, we both measured our weights. I have lost 4.5 kgs….. mostly when I have pity on her, I have stress. When you hear she is hurting, I feel sorry. I feel pain and I feel bad. ” CG10 35-year-old daughter-in law

“I used to farm pigs, chicken, but now I could not go on with it” CG9 62- year-old sister

Psycho-social and physical needs related to impact of caregiving was observed among some informal caregivers. End-of life care needs, information needs, and coping strategies remained largely unaddressed among many informal caregivers of advanced cancer patients:

“I was scared because I was told grandpa’s life is about to end. I just felt scared. I just felt… I just say like he had departed...... and he is nearing the end…… Someone once you’ve spent time with them then you are told their life is in trouble- you go into denial” CG8 18-year-old grandson

One time I was very sick. I was in tears; my body was weak. I had even gone to Gatundu level 5, and the doctor did some tests on me. He told me that I was overwhelmed by stress and told me not to think a lot… I was just crying by myself, I couldn’t cry were my kids or husband were CG6 37-year-old wife

“But im here like a prisoner. When iam told to go to work as a mason, I cannot go far. I must stay close. I have lost many job opportunities” CG5 67-year-old wife

The informal caregivers’ other tasks also compromise the time and care provided to other children. In one visit, we found a seven-year-old child with a dirty burn, which she sustained trying to get porridge out of the fire. One difficulty the informal caregivers expressed was helping their children to deal with emotions that arose from watching the patient deal with pain, inability to eat, vomiting and other symptoms. Caregiver 8 who had 7 children between the ages of 4 years and 17 years reported:

“Even the children were stressed from the eldest to the smallest. All of them. Even the teachers were asking me what is wrong with the children. I told them (the teachers) to let them be because they are seeing the way their father is in pain. Because they saw him vomiting after eating anything. I don’t know what to do with the children.” CG 6 37-year-old wife

One caregiver was taking care of two patients - his grandfather with prostate cancer with spine metastasis and his grandmother with a spinal cord injury.

“For me, I serve two masters…. In addition to the farm work, I take care of guka (grandfather) who has cancer (prostate cancer with spine metastasis and paraplegia) and cucu (grandmother) who had a spinal cord injury and even she cannot walk” CG 8 18-year-old grandson

A respondent aged 42 taking care of 2 children under 4 and her husband with metastatic lung disease reported:

“I have to be up by 3 am to milk the cows…. Then I come to help the children prepare for school” CG 7 42-year-old wife

Another 35-year-old respondent taking care of her mother-in-law with esophageal cancer and 3 school-going children reported:

“I wake up at 5 30, prepare her (patient’s) porridge, and prepare breakfast for the children, make sure they take it before they go to school” CG 10 35-year-old daughter in law

The tasks that informal caregivers did for their patients varied with the dependence of the patient. They mostly included feeding the patient, bathing the patient, laundry, helping with toilet functions, dressing wounds, administering medication, lifting the patients and turning them, taking them outside to sun-bask. Other mental roles include deciding on when the patient required attention and where to take them. One caregiver described that all these tasks made him feel that he was a prisoner in his own compound because he couldn’t go far at any given time. One 53 year old respondent giving care to his father who was paralyzed secondary to metastatic prostate cancer reported:

“…. And you see even if it’s going out, I cannot go out for long hours, Because I have to know the time for his medication and be present then. CG 1 53-year-old son

Socio-economic needs and financial trapping trajectory related to treatment and management of cancers at advanced stage also created unlimited economic needs among all informal caregivers. Majority experienced difficulties of having to face expensive or costly treatments with limited resources or a times unhelpful state-cover insurance (NHIF) and hence found disposing of assets and good the only option and way out. Other informal caregivers were in state of helplessness due to inability to afford means to transport their informal caregivers for treatment.

“I had cows that I have since sold one after the other so that I do not disturb my children. I have sold everything that I had. Goats, cows, everything is over because of this sickness because I have spent a lot of money……I thought NHIF would help me, but it has not helped me much because have still sold my property ” CG5 67-year-old husband

“The road is not good for cars to pass through. So she uses a motorbike upto where the car is, and the journey is usually difficult for her. Because you see when we are using a matatu (public means) it stops at all stages and it makes her very dizzy and uncomfortable…. And we cant afford a private care. “ CG4 26 year-old daughter

“Even I do not sleep in…..normally I sleep here on the couch so that when he calls I can hear…. He calls out so that he may be turned in bed, or so that I massage his legs” CG2 21-year-old grandson

“I have been living most of the time in stress. Sometimes you find that I cannot eat because of stress. When he is in a lot of pain, the moment he is in a lot of pain, I am stressed……I get angered easily” CG1 53-year-old son

“My children are not financially capable, so they have to rely on me. And I still have to take care of my wife. So my main challenge now is financial. And I don’t have a house help to care for her, I do not have the money to hire one. “ CG567-year-old husband

The physical effects ranged from eating disturbances with accompanying weight loss and sleep disturbances. One caregiver described actually feeling physical pain when she sees her loved one in pain. Caregiver 6 spoke of a time when she physically felt unwell, and required hospitaladmission before they realized that she was simply overwhelmed, and had counseling prior to discharge. Caregiver 12 opted to postpone her own healthcare needs. She was scheduled for a total hip replacement due to severe right hip osteoarthritis-that even affected her mobility in the house. She “chose” to postpone her care because “she did not have time” and the “resources” they had were being channeled towards the husband’s health.

One 18-year-old respondent taking care of his grandfather who was paralyzed secondary to metastatic pancreatic cancer reported:

“I normally don’t sleep well, I just sleep here on the couch, so that I can be able to respond to him when he calls” CG 2 21-year-old grandson

Another caregiver supporting her sister through chemotherapy stated:

“During this period, I have lost about 5 kgs…I think it’s because I am not eating. It’s hard to eat when she doesn’t eat.” CG 9 62-year-old sister

The mental and psychological effects were due to anxieties of what next, should the patient die. Occasionally, it was due to anticipated grief. One caregiver reported:

“I can say I have been most affected psychologically because I wonder what next. I wonder when I have gone to work and left him if he will still be there. When I find him in the bedroom I tell God thank you ” CG 3 41-year-old daughter

“I was stressed because, in Kenyatta, I was told that he would die, and even he (the patient) seemed to agree….” CG6 37-year-old wife

It’s also imperative to consider the impact on the children as well. If the caregiver has parenting responsibilities, they need to be equipped on how to care for the children, as they too feel the stress, and can get overwhelmed. This is depicted in the excerpt below.

“Even the teachers were asking me what is wrong with the children…….they regressed in their studies.” CG 637-year-old wife

## Discussion

Informal caregivers play a key role in palliative care in sub-Saharan Africa, a context fraught with inadequate healthcare services coupled with poverty, making home-based care the only available option for many chronically and terminally ill persons [21]. The significance of the caregiver role is indicative of the need to support the informal caregivers’ well-being.

As the disease progressed, the level of patient dependence increased, and so does the demands on the caregiver. The other competing realities of life like farm work, childcare and other social responsibilities remained, and no provision was made to adjust effects of the impact to informal caregivers of cancer patients. More studies are required to explore changing caregiver needs and support network

Increasing unmet needs among informal caregivers have been worsened by the fact that there is no recognition and support to informal health caregivers. A study has shown that caregiver labor costs were often unappreciated by their informal caregivers and completely unrecognized by the healthcare system at large [22]. This is similar to our setting where caregivers’ contribution to the informal healthcare workforce goes unrecognized and uncompensated by the government and the healthcare system. There was no alternative option to support the caregiver. It is thought that hospice support and respite care to support caregivers are a luxury of the rich in Kenya. There is therefore need to re-look at the community-based level support needs in collaboration with hospice staff in Kenya and increasing awareness of the available support from palliative care team in rural parts where the majority of patients reside.

In this study, informal caregivers faced enormous challenges related to unpreparedness to handle end-of-life care for the patients in their advanced stage of cancers. The majority were strained, stressed and felt helpless in the face of impending death of their loved ones. Though informal caregivers were resilient, they were overwhelmed with their caregiving role amidst other competing tasks, had increasing unmet needs which negatively impacted on their quality of life. One study has mentioned up to thirty different tasks that informal caregivers are involved in, the majority of which our informal caregivers shared [23]. Tasks included medication acquisition and dispensing, symptom management, meals and nutritional assistance, supervision of treatments, adherence, emotional support, communication with the healthcare providers, and many others. There is urgent need to address gaps and informational needs related to end of life care among caregivers. This study strengthens the need to raise awareness and equip informal caregivers with requisite knowledge and skills to face end-of life. This study provides sufficient evidence and basis to inform future practice of palliative and end of life care among informal caregivers facing complex coordination of tasks and logistics required to deliver needed care.

Unmet needs worsened by role-related strain experienced by informal caregivers was evident in this study. In other studies, role strain correlates to the number of other competing roles, which in turn is determined by the stage of lifespan that a caregiver was in [1, 3] Erdwins and his colleagues had findings that supported the role-strain theory: that the more social roles a caregiver carries out, the more likely the caregiver is to experience stress, strain and negative affect [24]. This implies that for informal caregivers who also had to care for their own children and other farm work would, therefore, be expected to experience greater role-strain, and potentially be more overwhelmed, than caregivers who simply had one caregiving task alone. While there were no objective assessments done to assess informal caregivers’ burden, it did seem true in this study that informal caregivers, particularly those that had school going children experienced a greater burden contributing to increased unmet needs. Further studies is need to understand the dynamics, context of caregiving in African-setting where some caregivers double up as breadwinners in the family and whose role gets severely affected by the new role of caregiving. Studies into how best to support caregivers with basic necessities is necessary.

The study showed that there are a lot of unaddressed issues for further investigation along primary caregiving experiences which merit inquiry. For instance, mainstreaming palliative care (patient and informal caregivers) into healthcare system in all health care facilities in Kenya. This study revealed lack of priorities and ways of integrating informal caregivers into formal health care settings. Other studies have demonstrated similar need to explore research and clinical priorities for informal cancer caregiving and giving recommendations for moving science forward [4]. Study findings serve as drivers for future research on ways and approaches to improve estimation of the prevalence and burden of informal cancer caregiving, advancing the development of interventions designed to improve outcomes for patient-caregiver dyads, generating and testing strategies for integrating informal caregivers into formal health care settings; and promoting the use of technology to support informal cancer caregivers have been documented [4, 25]. Further study is needed at government-facilities offering palliative care services in Kenya to explore preparedness, resilience, and unmet needs of informal caregivers of advanced cancer patients. This study limitation include inability to generalize its findings. Another limitation of this study includes settings; it was done within mission-based referral hospital which may not represent the true health care system in Kenya

### Recommendations

There is dire need to re-look at the support and changing needs of informal caregivers and address them. Palliative care teams should be proactive to form support groups where informal caregivers can share their challenges and be of encouragement to each other. A caregiver- tailored screening tool could be introduced to assess prevalence and burden of caregiving, level of preparedness and needs as well as the impact on the caregiver and their ability to care for their patients. This would help healthcare workers assess the informal caregivers’ willingness and ability to offer quality end of life care and any further interventions that may be of benefit to the caregiver. The family debriefing meetings should be done with the primary caregiver and other support persons where necessary so that they can all be on board with the patient and primary caregiver needs. The debriefing meetings should include a session/talk on how the other informal caregivers should support the primary caregiver.

## Data Availability

The datasets used and/or analysed during the current study available from the corresponding author on reasonable request.
